# Two-sample Mendelian randomization to study the causal association between gut microbiota and atherosclerosis

**DOI:** 10.3389/fimmu.2023.1282072

**Published:** 2024-01-12

**Authors:** Shijiu Jiang, Cheng Yu, Bingjie Lv, Shaolin He, Yuqi Zheng, Wenling Yang, Boyuan Wang, Dazhu Li, Jibin Lin

**Affiliations:** ^1^ Department of Cardiology, Union Hospital, Tongji Medical College, Huazhong University of Science and Technology, Wuhan, China; ^2^ Hubei Key Laboratory of Biological Targeted Therapy, Union Hospital, Tongji Medical College, Huazhong University of Science and Technology, Wuhan, China; ^3^ Hubei Provincial Engineering Research Center of Immunological Diagnosis and Therapy for Cardiovascular Diseases, Union Hospital, Tongji Medical College, Huazhong University of Science and Technology, Wuhan, China; ^4^ Department of Cardiology, The First Affiliated Hospital, Shihezi University, Shihezi, China; ^5^ Department of Neurology, Union Hospital, Tongji Medical College, Huazhong University of Science and Technology, Wuhan, China

**Keywords:** cerebral atherosclerosis, coronary atherosclerosis, gut microbiota, Mendelian randomization, peripheral atherosclerosis

## Abstract

**Background:**

According to some recent observational studies, the gut microbiota influences atherosclerosis via the gut microbiota-artery axis. However, the causal role of the gut microbiota in atherosclerosis remains unclear. Therefore, we used a Mendelian randomization (MR) strategy to try to dissect this causative link.

**Methods:**

The biggest known genome-wide association study (GWAS) (n = 13,266) from the MiBioGen collaboration was used to provide summary data on the gut microbiota for a two-sample MR research. Data on atherosclerosis were obtained from publicly available GWAS data from the FinnGen consortium, including cerebral atherosclerosis (104 cases and 218,688 controls), coronary atherosclerosis (23,363 cases and 187,840 controls), and peripheral atherosclerosis (6631 cases and 162,201 controls). The causal link between gut microbiota and atherosclerosis was investigated using inverse variance weighting, MR-Egger, weighted median, weighted mode, and simple mode approaches, among which inverse variance weighting was the main research method. Cochran’s Q statistic was used to quantify the heterogeneity of instrumental variables (IVs), and the MR Egger intercept test was used to assess the pleiotropy of IVs.

**Results:**

Inverse-variance-weighted (IVW) estimation showed that *genus Ruminiclostridium 9* had a protective influence on cerebral atherosclerosis (OR = 0.10, 95% CI: 0.01–0.67, *P* = 0.018), while *family Rikenellaceae* (OR = 5.39, 95% CI: 1.50–19.37, *P* = 0.010), *family Streptococcaceae* (OR = 6.87, 95% CI: 1.60–29.49, *P* = 0.010), *genus Paraprevotella* (OR = 2.88, 95% CI: 1.18–7.05, *P* = 0.021), and *genus Streptococcus* (OR = 5.26, 95% CI: 1.28–21.61, *P* = 0.021) had pathogenic effects on cerebral atherosclerosis. For *family Acidaminococcaceae* (OR = 0.87, 95% CI: 0.76–0.99, *P* = 0.039), the *genus Desulfovibrio* (OR = 0.89, 95% CI: 0.80–1.00, *P* = 0.048), the *genus RuminococcaceaeUCG010* (OR = 0.80, 95% CI: 0.69–0.94, *P* = 0.006), and the *Firmicutes phyla* (OR = 0.87, 95% CI: 0.77–0.98, *P* = 0.023) were protective against coronary atherosclerosis. However, the *genus Catenibacterium* (OR = 1.12, 95% CI: 1.00–1.24, *P* = 0.049) had a pathogenic effect on coronary atherosclerosis. Finally, *class Actinobacteria* (OR = 0.83, 95% CI: 0.69–0.99, *P* = 0.036), *family Acidaminococcaceae* (OR = 0.76, 95% CI: 0.61–0.94, *P* = 0.013), *genus Coprococcus2* (OR = 0.76, 95% CI: 0.60–0.96, *P* = 0.022), and *genus RuminococcaceaeUCG010* (OR = 0.65, 95% CI: 0.46–0.92, *P* = 0.013), these four microbiota have a protective effect on peripheral atherosclerosis. However, for the *genus Lachnoclostridium* (OR = 1.25, 95% CI: 1.01–1.56, *P* = 0.040) and the *genus LachnospiraceaeUCG001* (OR = 1.22, 95% CI: 1.04–1.42, *P* = 0.016), there is a pathogenic role for peripheral atherosclerosis. No heterogeneity was found for instrumental variables, and no considerable horizontal pleiotropy was observed.

**Conclusion:**

We discovered that the presence of probiotics and pathogens in the host is causally associated with atherosclerosis, and atherosclerosis at different sites is causally linked to specific gut microbiota. The specific gut microbiota associated with atherosclerosis identified by Mendelian randomization studies provides precise clinical targets for the treatment of atherosclerosis. In the future, we can further examine the gut microbiota’s therapeutic potential for atherosclerosis if we have a better grasp of the causal relationship between it and atherosclerosis.

## 1 Introduction

Gut microbiota are microorganisms that colonize the host gut and may affect host physiology in various ways. Increasing evidence suggests that dysregulation of the gut microbiota is associated with the pathogenesis of various cardiovascular diseases (CVD), such as atherosclerosis, heart failure, atrial fibrillation, hypertension, obesity, and dyslipidemia (1). Atherosclerosis (AS), which is characterized by lipid accumulation and immune-inflammatory changes in arterial vessels, is a major contributor to CVD and may eventually result in its clinical complications, including cerebrovascular accident, myocardial infarction, and peripheral artery embolism (2). Since the development of AS is regulated by the gut microbiota (GM) and its metabolites, scholars regard this regulation mode as the GM arterial regulation axis (2). Gut microbiota plays a role in atherosclerosis mainly in the following three ways (3): First, the infection of the gut microbiota may lead to a harmful immune inflammatory response, thereby aggravating the formation of plaque or triggering plaque rupture. Secondly, the regulation of lipid metabolism by the gut microbiota affects the progression of atherosclerotic plaques. Finally, specific components of diet and gut microbiota metabolism can have multiple effects on atherosclerosis; for example, dietary fiber is beneficial for AS, whereas trimethylamine-N-oxide (TMAO), a metabolite of gut microbiota, is thought to be detrimental.

In addition, specific commensal bacteria in the host can be protective against AS. However, pathogens or opportunistic pathogens can promote atherosclerosis. Both types regulate host metabolism and inflammatory responses directly or indirectly via their metabolites (4). For example, earlier research has established that *Akkermansia muciniphila* and *Lactobacillus* may be next-generation probiotics or live biotherapeutic products that can reduce the risk of AS (2). Treatment with Akkermansia muciniphila reduces macrophage infiltration, chemokines, and pro-inflammatory cytokines and protects the integrity of the intestinal barrier, thereby alleviating AS lesions (5). In addition, several studies have shown that alterations in the gut microbial composition in obese patients are associated with the progression of AS (6, 7), the most obvious changes were the decrease in the proportion of *Bacteroidetes phylum* and the increase in the proportion of *Firmicutes phyla*. In addition, pathogen and opportunistic pathogens including *Actinomycetes, Porphyromonas gingivalis, aggregating bacilli*, *Streptococcus hemolytic, Streptococcus pneumoniae, Staphylococcus aureus, Streptococcus viridans*, etc., which promote the transport of intestinal bacteria by destroying the integrity of the intestinal barrier and promoting the formation of atherosclerotic plaques, are considered to promote AS (8, 9).

In addition to the pathogenic role of their pathogens, gut microbes can also affect the process of atherosclerosis through their metabolites. The well-known metabolites are TMAO (10), secondary bile acids (11), short-chain fatty acids (12), and lipopolysaccharide (13) are also involved in the process of atherosclerosis. For instance, Synphytes, Clostridium, Desalinobacter, Desulfurvibrio, and members of Fusobacteriaceae have been linked to the development of AS by significantly positive correlations with TMAO (14).

Despite the rise in research linking GM and AS, it’s crucial to remember that a correlation does not imply a cause-and-effect relationship. Due to possible biases including confounding and reverse causality, as well as the fact that the majority of previous research were case-control studies, it is uncertain whether these correlations are causal. Additionally, in observational research, confounding variables such as dietary patterns, age, environment, and lifestyle are easily able to influence the relationship between gut microbiota and AS (15).

Mendelian randomization (MR), a trustworthy technique for examining causal relationships, employs genetic variations as instrumental variables (IVs) to ascertain if exposure and outcome are causally related (16). Given that genotypes are randomly assigned from parents to children, common confounding variables have little impact on the relationship between genetic variation and outcome, and the causal chain is reliable (17). However, no research has utilized MR analysis to identify potential causal relationships between the gut microbiota and the risk of atherosclerosis. As a result, MR analysis was employed in this study to completely examine the potential that the gut microbiota and AS are causally related and to uncover certain pathogenic or therapeutic bacterial communities.

## 2 Methods

### 2.1 Design of the study

Throughout the study, we adhered to the principles outlined in the STROBE-MR Statement for reporting observational studies in Epidemiology (18).

Data from prior research’ published genome-wide association studies (GWAS) were used in this MR analysis. The authors of the GWAS database obtained the relevant ethics and institutional review board authorizations and participant consents to permit their studies. Therefore, our MR analysis from published and anonymized data did not need further ethical approval. In this study, a GWAS summary dataset was used to evaluate the causal relationship between gut microbiota and AS, and a heterogeneity test and sensitivity analysis were carried out to ensure the reliability of the results.

**Figure 1:**
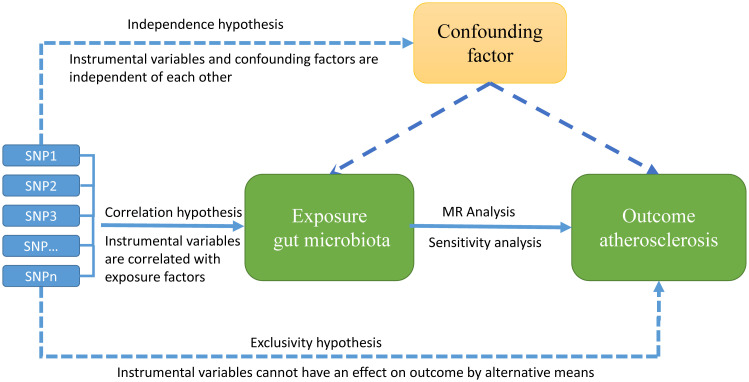
Overview of MR hypotheses, design, and procedures. There are three key hypotheses for MR study. hypotheses 1: Instrumental variables (IVs) must be strongly correlated with exposure factors; hypotheses 2: the used IVs should not be associated with any potential confounder; hypotheses 3: the IVs should influence the outcome risk merely through the exposures, not via any alternative pathway.

An MR study needs to satisfy three core hypotheses: the correlation hypothesis, the independence hypothesis, and the exclusivity hypothesis, namely: 1. Exposure factors and instrumental variables (IVs) must be closely connected; 2. IVs cannot be correlated with any confounding variables related to the expose-outcome relationship; 3. IVs can only impact outcome variables through exposure factors (**Figure 1**).

### 2.2 GWAS summary data sources

The GWAS project opened by the IEU in 2021, which provides the largest published GWAS summary statistics on atherosclerosis, was selected for this study. GWAS data for AS were obtained from publicly available GWAS data from the FinnGen consortium, including cerebral atherosclerosis (104 cases and 218,688 controls), coronary atherosclerosis (23,363 cases and 187,840 controls), and peripheral atherosclerosis (6631 cases and 162,201 controls).

The GWAS summary microbiota statistics were mainly obtained from MiBioGen Consortium (www.mibiogen.org), 18,340 participants from 24 cohorts were included, 211 taxonomic units were recorded (35 families, 29 orders, 16 phyla, 131 genera) and 122, 110 associated SNPs (19–21), the detailed data sources showed in **Table 1**.

**Table 1:** Characteristics of GWAS data for gut microbiota traits and Atherosclerosis.

**Trait**	**Sample size**	**Consortium**	**Link**	**Year**
Gut microbiota	18,340	MiBioGen	https://mibiogen.gcc.rug.nl/	2021
Coronary atherosclerosis	211,203	FinnGen	https://gwas.mrcieu.ac.uk/datasets/finn-b-I9_CORATHER/	2021
Cerebral atherosclerosis	218792	FinnGen	https://gwas.mrcieu.ac.uk/datasets/finn-b-I9_CEREBATHER/	2021
Peripheral atherosclerosis	168832	FinnGen	https://gwas.mrcieu.ac.uk/datasets/finn-b-DM_PERIPHATHERO/	2021

GWAS, genome-wide association studies.

### 2.3 Selection and verification of IVs

First, to satisfy the first MR hypothesis that single-nucleotide polymorphisms (SNPs) need to be tightly connected to gut microbiota, SNPs that were highly related to gut microbiota were chosen at the genome-wide level (linkage disequilibrium [LD], r^2^ < 0.001, genome-wide significance threshold < 1×10^−5^, genetic distance = 10,000 kb) (20). Second, to ensure that the second MR hypothesis, that genetic variation is not associated with potential confounding factors, we examined the phenoscanner database (22) to determine that the included SNPs were not associated with known confounding factors, such as smoking status, blood pressure, sex, family history of hypertension, dyslipidemia, diabetes, and body mass index (BMI). A heterogeneity test was used to eliminate significantly heterogeneous SNPs, and SNPs substantially linked with gut microbiota were discovered as IVs.

Palindromic SNPS may also contribute to bias in the estimate of causation (21), because the alleles of the two palindromic SNPS are not independent and may violate the MR Hypothesis. We removed palindromic SNPS from instrumental variables to ensure the validity of the results and to increase confidence in causal inference.

The F statistic is calculated to evaluate whether the selected IVs are weak. F > 10 indicates that there are no weak IVs to further verify the relevance hypothesis. The computation algorithm is F =β^2^_exposure_/SE^2^_exposure_, it is estimated according to beta and standard error. The strength of the connection between the IVs and the exposure phenotype was assessed using the F statistic; SNPs with an F-statistic < 10 should be disregarded (23). The traits of the genetic IVs for gut microbiota are listed below (**Supplementary Table 1**).

### 2.4 MR analysis

To better assess the full causal connection between gut microbiota and AS, a two-sample MR analysis was performed using IVW as the main analysis method, four more complimentary analytic techniques (MR Egger, simple mode, weighted median, and weighted mode) were also employed. In addition, a threshold of *P <*0.05 was used to represent a significant causal relationship between gut microbiota and AS.

### 2.5 Pleiotropy test and heterogeneity test

First of all, the MR-PRESSO method (24) was used to detect outliers in this study. If there were outliers, they would be removed and re-analyzed. The “leave one out” sensitivity analysis (25) was carried out by removing individual SNPs at a time to assess whether the IVs drove the association between exposure and outcome. Secondly, to make it clear whether the MR analysis has horizontal polymorphism, the MR-Egger intercept item (26) is also detected in this study. If the intercepted item in the MR-Egger intercept analysis has obvious statistical significance, it indicates that the study has obvious horizontal polymorphism. Finally, this study also uses Cochran’s Q measurement to test heterogeneity, it may demonstrate heterogeneity brought on by pleiotropy and other uncertain factors. IVW and MR-Egger in Cochran’s Q (27) statistics have been widely used to check heterogeneity. The results of the test for pleiotropy and heterogeneity are shown in the **supplementary table**. *P* > 0.05 indicated no significant pleiotropy or heterogeneity.

## 3 Results

### 3.1 Selection of IVs

After a series of quality controls for cerebral atherosclerosis, we extracted 65 independent SNPs (*P* < 1.0×10^−5^, r^2^<0.001) associated with five bacterial genera as IVs. For coronary atherosclerosis, we extracted 41 independent SNPS associated with 5 bacterial genera as IVs, and for peripheral atherosclerosis, we extracted 62 independent SNPs associated with 6 bacterial genera as IVs; all IVs had F statistics greater than 10, indicating that the selected SNPs all had sufficiently strong IVs effects without weak IVs bias. The results of the IVs association between AS and gut microbiota were detailed in **Supplementary Tables 1, 5, 9**.

MR_egger and IVW in Cochran’s Q test both showed no significant heterogeneity in the genetic IVs associated with cerebral atherosclerosis, coronary atherosclerosis, and peripheral atherosclerosis (**Supplementary Tables 3, 7, 11**). In addition, the MR-egger intercept test showed that there was no significant pleiotropy of the genetic IVs related to cerebral atherosclerosis, coronary atherosclerosis, and peripheral atherosclerosis (*P* > 0.05). The results were detailed in **Supplementary Tables 4, 8, 12**. Therefore, the genetic IVs of all selected gut microbiota should be considered valid IVs in this MR analysis.

### 3.2 MR analysis

**Figure 2:**
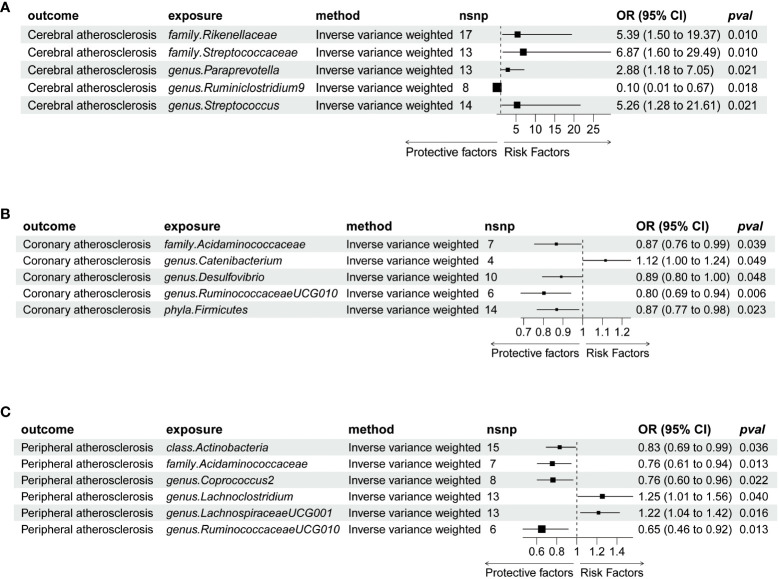
Forrest plot for summary causal effects of gut microbiota on atherosclerosis risk based on MR methods (inverse variance weighted IVW); **(A)** Represents the causal effect of gut microbiota on cerebral atherosclerosis. **(B)** Represents the causal effect of gut microbiota on coronary atherosclerosis. **(C)** Represents the causal effect of gut microbiota on peripheral atherosclerosis. MR, Mendelian randomization; nSNP, number of single-nucleotide polymorphism; OR, odds ratio.

**Figure 3:**
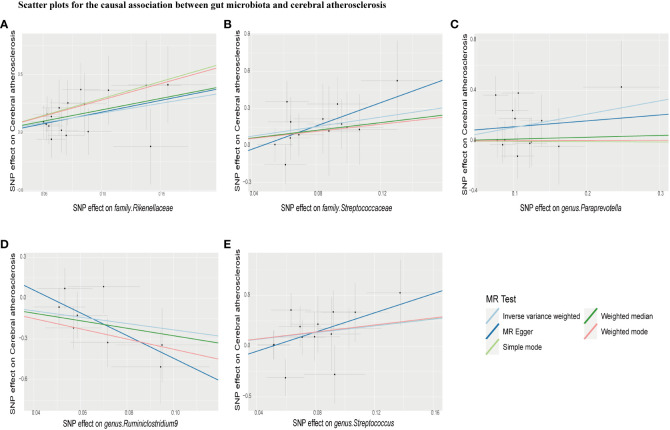
Scatter plots for causal effects of gut microbiota on cerebral atherosclerosis risk using five MR methods. **(A–E)** Represents the causal effects of family.Rikenellaceae, family.Streptococcaceae, genus.Paraprevotella, genus.Ruminiclostridium9, and genus. Streptococcus on cerebral atherosclerosis, respectively.

**Figure 4:**
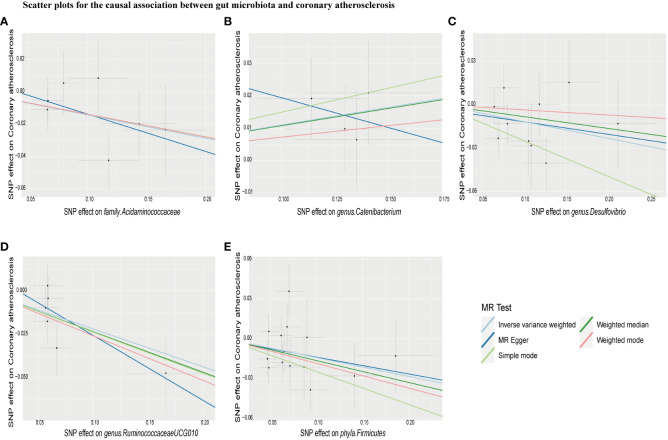
Scatter plots for causal effects of gut microbiota on coronary atherosclerosis risk using five MR methods. **(A–E)** Represents the causal effects of family.Acidaminococcaceae, genus.Catenibacterium, genus.Desulfovibrio, genus.RuminococcaceaeUCG010, and phyla.Firmicutes on coronary atherosclerosis, respectively.

**Figure 5:**
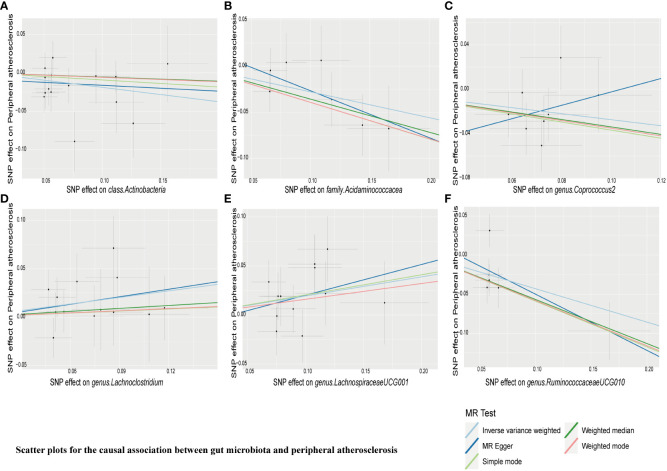
Scatter plots for causal effects of gut microbiota on peripheral atherosclerosis risk using five MR methods. **(A–F)** Represents the causal effects of class.Actinobacteria, family.Acidaminococcacea, genus.Coprococcus2, genus.Lachnoclostridium, genus.LachnospiraceaeUCG001, and genus.RuminococcaceaeUCG010 on peripheral atherosclerosis, respectively.

IVW estimation showed that *genus Ruminiclostridium 9* had a protective effect on cerebral atherosclerosis (OR = 0.10, 95% CI: 0.01–0.67, *P* = 0.018), while *family Rikenellaceae* (OR = 5.39, 95% CI: 1.50–19.37, *P* = 0.010), *family Streptococcaceae* (OR = 6.87, 95% CI: 1.60–29.49, *P* = 0.010), *genus Paraprevotella* (OR = 2.88, 95% CI: 1.18–7.05, *P* = 0.021), and *genus Streptococcus* (OR = 5.26, 95% CI: 1.28–21.61, *P* = 0.021) were pathogen and opportunistic pathogens to cerebral atherosclerosis (**Figures 2A**, **3**; **Supplementary Table 2**). As a causal inference for coronary atherosclerosis, we found *family Acidaminococcaceae* (OR = 0.87, 95% CI: (0.76–0.99, *P* = 0.039), *genus Desulfovibrio* (OR = 0.89, 95% CI: 0.80–1.00, *P* = 0.048), *genus RuminococcaceaeUCG010* (OR = 0.80, 95% CI: 0.69–0.94, *P* = 0.006), and *Firmicutes phyla* (OR = 0.87, 95% CI: 0.77–0.98, *P* = 0.023) were protective against coronary atherosclerosis. However, the *genus Catenibacterium* (OR = 1.12, 95% CI: 1.0–1.24, *P* = 0.049) had a pathogenic and opportunistic pathogenic effect on coronary atherosclerosis (**Figures 2B**, **4**, **Supplementary Table 6**). Finally, for the causal inference of peripheral atherosclerosis, we found that *class. Actinobacteria* (OR = 0.83, 95% CI: 0.69–0.99, *P* = 0.036), *family Acidaminococcaceae* (OR = 0.76, 95% CI: 0.61–0.94, *P* = 0.013), *genus Coprococcus 2* (OR = 0.76, 95% CI: 0.60–0.96, *P* = 0.022), *genus Ruminococcaceae UCG010* (OR = 0.65, 95% CI: 0.46–0.92, *P* = 0.013) for peripheral artery atherosclerosis has a protective effect. However, the *genus Lachnoclostridium* (OR = 1.25, 95% CI: 1.01–1.56, *P* = 0.040) and the *genus LachnospiraceaeUCG001* (OR = 1.22, 95% CI: 1.04–1.42, *P* = 0.016) had a pathogenic and opportunistic pathogenic effect on peripheral atherosclerosis (**Figures 2C**, **5**, **Supplementary Table 10**).

### 3.3 No significant bias in the effect of a single SNP in gut microbiota on AS

**Figure 6:**
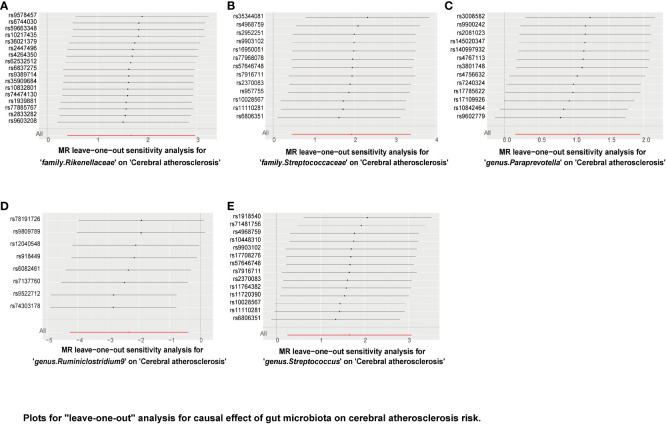
Plots for “leave-one-out” analysis for causal effect of gut microbiota on cerebral atherosclerosis risk. **(A–E)** Represents the MR leave−one−out sensitivity analysis for family.Rikenellaceae, family.Streptococcaceae, genus.Paraprevotella, genus.Ruminiclostridium9, and genus.Streptococcus on cerebral atherosclerosis, respectively.

**Figure 7:**
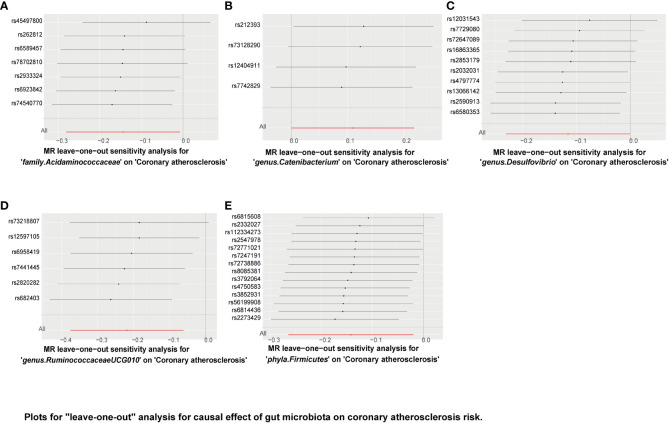
Plots for “leave-one-out” analysis for causal effect of gut microbiota on coronary atherosclerosis risk. **(A–E)** Represents the MR leave−one−out sensitivity analysis for family.Acidaminococcaceae, genus.Catenibacterium, genus.Desulfovibrio, genus.RuminococcaceaeUCG010, and phyla.Firmicutes on coronary atherosclerosis, respectively.

**Figure 8:**
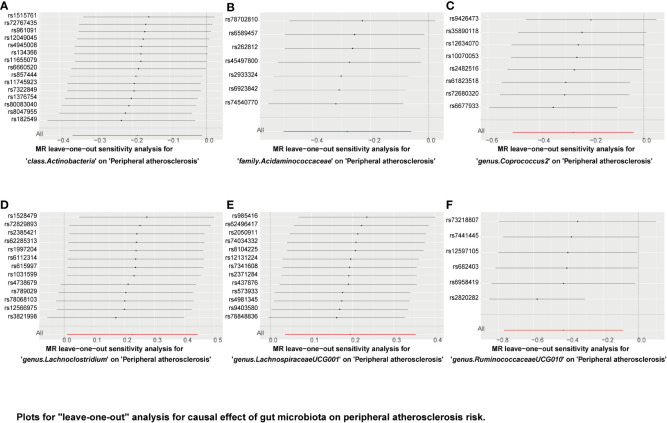
Plots for “leave-one-out” analysis for causal effect of gut microbiota on peripheral atherosclerosis risk. **(A–F)** Represents the MR leave−one−out sensitivity analysis for class.Actinobacteria, family.Acidaminococcacea, genus.Coprococcus2, genus.Lachnoclostridium, genus.LachnospiraceaeUCG001, and genus.RuminococcaceaeUCG010 on peripheral atherosclerosis, respectively.

“MR Leave-one-out” sensitivity analyses showed that the remaining SNPs after the removal of specific SNPs did not change the causal inference results (**Figures 6**–**8**), showing that no specific IVs were responsible for any of the found causal connections. Together, these results suggest that there is no significant bias in the effect of individual gut microbiota SNPs on atherosclerosis. In addition, we showed the causal effect of single SNPs by drawing forest plots, and the results showed that the effect of single SNPs was consistent with the results of the combined effect of IVW (**Supplementary Figures 1**–**3**).

## 4 Discussion

In this work, we performed two-sample MR analyses to determine the causative connection between gut microbiota and AS using summary statistics on gut microbiota from the biggest GWAS meta-analysis completed by the MiBioGen consortium and summary statistics on AS released by the FinnGen consortium. This study provides guidance for future research based on gut microbiota in the treatment of AS. As we all know, resident microbial communities in the gut are key “metabolic filters” in the diet, as these species can convert common nutrients into metabolites, and specific microbiota-associated metabolites, such as TMAO, short-chain fatty acids (SCFAs), and secondary bile acids, have been shown to influence CVD progression (28–32).

We found that specific gut microbiota may be causally linked to AS at different sites. For example, the *genus Ruminiclostridium 9* is negatively associated with the risk of cerebral atherosclerosis and has a protective effect. However, the *family Rikenellaceae*, the *family Streptococcaceae*, the *genus Paraprevotella*, and the *genus Streptococcus* are significantly linked to the risk of cerebral atherosclerosis. Therefore, These four intestinal microbes may contribute to the pathogenesis of cerebral atherosclerosis. in the family. *Acidaminococcaceae* and the *genus Ruminococcaceae (UCG010)* both have protective effects on coronary atherosclerosis and peripheral atherosclerosis. The *genus Desulfovibrio* and the *Firmicutes phyla* were specifically negatively associated with coronary atherosclerosis risk and had a protective effect, while the *genus Catenibacterium* was positively associated with coronary atherosclerosis risk and had an atherogenic effect. *class Actinobacteria* and *genus Coprococcus 2* are specifically negatively correlated with the risk of peripheral atherosclerosis and have a protective effect. *Genus Lachnoclostridium* and *genus Lachnospiraceae UCG001* specificity increased the risk of peripheral artery atherosclerosis. Our research will contribute to the theoretical foundation for AS precision therapy in the future. As atherosclerosis in different sites is associated with specific microbiota, we hypothesize that this may be because specific metabolites of the microbiota are related to the microenvironment of different arterial locations.

In addition, we also found that *Acidaminococcaceae* and *Ruminococcaceae UCG010* have the same protective effect on coronary atherosclerosis and peripheral atherosclerosis, and *Ruminiclostridium 9* has the same protective effect on cerebral atherosclerosis. Oxana M. Drapkina (33) evaluated the impact of fecal microbiota on atherosclerotic cardiovascular disease (ASCVD) and heart failure with reduced ejection fraction (HFrEF) by using bacterial culture, 16S next-generation sequencing (NGS) of the 16S rRNA gene (V3-V4), and quantitative polymerase chain reaction (qPCR). They found that *acidaminococcaceae* were significantly lower in the ASCVD and HFrEF groups, and *acidaminococcaceae* were negatively associated with ASCVD. while *Streptococcaceae* were significantly increased in ASCVD and HFrEF groups. In our study, we found that *acidaminococcaceae*, one of the commensal bacteria with an atherogenic effect, was also negatively associated with cerebral atherosclerosis and peripheral atherosclerosis. Kesavalu L (34) found that *Streptococcus mutans* infection accelerated plaque growth, macrophage invasion, and TLR4 expression after angioplasty, and *Streptococcus mutans* may also be associated with atherosclerotic plaque growth in noninjured arteries. Koren et al (35) identified Veloxella and *Streptococcus* in AS plaque samples, and several bacterial types in the gut are common in atherosclerotic plaques and correlated with cholesterol levels. Another metagenomic association study (36) showed that the abundance of *Streptococcus* in patients with atherosclerotic cardiovascular disease was higher than that in the healthy control group. Therefore, *Streptococcaceae* is considered a pathogenic bacterium and can increase the risk of atherosclerosis, which is consistent with our findings.

In addition, according to this study, the abundance of *Ruminiclostridium* in the heart failure with preserved ejection fraction (HFpEF) group was lower than that in the control group, Qiuxia Liu (37) also found that the relative abundance of *Ruminococcaceae* was positively correlated with the level of HDL through 16S ribosomal DNA sequencing. Therefore, *Ruminococcaceae* can inhibit atherosclerosis, consistent with our findings. *Ruminiclostridium 9* can alleviate the formation of cerebral atherosclerosis, and *Ruminococcaceae UCG010* can inhibit the formation of coronary and peripheral atherosclerosis. Hannelore Daniel’s study (38) found that a high-fat diet caused shifts in the diversity of dominant gut bacteria and altered the proportion of *Ruminococcaceae* (decrease) and *Rikenellaceae* (increase). Our results suggest that *Rikenellaceae* can increase the risk of cerebral atherosclerosis, while *Ruminococcaceae* are negatively correlated with coronary atherosclerosis and peripheral atherosclerosis. A recent study included in the TwinsUK cohort showed that (39) *Ruminococcaceae* was negatively correlated with pulse wave velocity (PWV), which represents arterial stiffness. *Ruminococcaceae* is a bacterium that can produce butyrate, and the increase in its abundance can reduce the release of inflammatory factors and alleviate endothelial dysfunction, thus delaying the development of atherosclerosis. Our research results also support the idea that *Ruminococcaceae* belongs to the probiotic family. Additional randomized controlled studies, nevertheless, are necessary to verify these results.

Omry Koren (35) found that the atherosclerotic plaques contained significantly fewer *Firmicutes phyla* and suggested a negative correlation with the risk of atherosclerosis; low intestinal levels were associated with greater risk, whereas normal or elevated levels were protective. Some studies have shown that butyrate is usually produced by *Firmicutes phyla* (40). If the *Firmicutes phyla* population is reduced, the concentration of butyrate in the intestine will decrease, leading to reduced mucin synthesis, and a lack of sufficient mucin on the intestinal membrane will lead to increased intestinal permeability (41), which induces a chronic inflammatory state, leading to a higher intestinal inflammatory state. These included increased concentrations of IL-1 and IL-4. IL-1 is a proinflammatory cytokine (42), which is associated with atherogenesis, plaque instability, plaque rupture, and thrombosis, and increases cardiovascular risk. Therefore, *Firmicutes phyla* belong to commensal bacteria and can inhibit arterial atherosclerosis, and our results also support the role of *Firmicutes phyla* in inhibiting atherosclerosis.

Akihiro Nakajima (43) found *paraprevotella* had a positive correlation with fibrinogen in plaque and a negative correlation with high-density lipoprotein cholesterol; *paraprevotella* were also associated with greater plaque volume. Our study also found that *paraprevotella* could promote the formation of cerebral atherosclerosis, which is consistent with our study results.

The research work of Yuan-Yuan Cai (44) provided a comprehensive metagenomic analysis of bacteria producing TMA (the precursor of TMAO) in the human gut and reported the *genus Lachnoclostridium* producing TMA for the first time. The abundance of this genus was higher in patients with atherosclerosis compared to healthy controls. They found *in vitro* that *Lachnoclostridium* can produce TMA when incubated with choline. *In vivo* studies further demonstrated that *Lachnoclostridium* could promote TMAO levels in the serum of ApoE^−/−^ mice, significantly elevate aortic plaque, and accelerate plaque formation *in vivo*. Therefore, targeting *Lachnoclostridium* may serve as a potential therapeutic target for the treatment of atherosclerosis. Our findings are consistent with those of the present study, suggesting that *Lachnoclostridium* promotes atherogenesis.

In addition, we also found some new probiotics whose effects on AS have not been reported before; for example, *Coprococcus 2* and *Actinobacteria* have protective effects on AS, and their specific protective mechanisms still need to be further explored. They can be used as a new therapeutic target for anti-atherosclerosis. Of note, *Desulfovibrio* suggested a negative association with coronary atherosclerosis in our study; However, in addition, we also found some new probiotics whose effects on AS have not been reported before; for example, *Coprococcus 2* and *Actinobacteria* have protective effects on AS, and their specific protective mechanisms still need to be further explored. They can be used as a new therapeutic target for anti-atherosclerosis. Of note, *Desulfovibrio* suggested a negative association with coronary atherosclerosis in our study; however, Kun Zhang’s results (45) demonstrate that D. desulfuricans can enhance the development of AS by increasing intestinal permeability and host inflammatory response, which is inconsistent with the results of our study, probably because we specifically targeted coronary atherosclerosis, while Kun Zhang’s research focused on aortic atherosclerosis, Different arterial sites have different microenvironments. There are also differences in the mechanisms of gut microbiota in AS. Therefore, more in-depth research mechanisms need to be further explored.

As far as we know, bacteria are a major component of the gut microbiome, but viruses, fungi, and archaea are also present, they live symbiotic in our gut. Although intestinal flora plays an important role in atherosclerosis, enteroviruses, fungi and their metabolites are also involved in the development of atherosclerosis (46, 47). First of all, the gut microbiota of adults is mainly composed of five phyla: *Bacteroidetes*, *Firmicutes*, *Actinobacteria*, *Proteobacteria*, and *Cerrucomicrobia* (48), and changes in the components of these flora can cause ecological imbalance of intestinal flora. Several studies have confirmed the presence of bacterial DNA in atherosclerotic plaques, thereby affecting plaque stability, which may contribute to the development of cardiovascular disease (49). The main pathogenic mechanism may be the impairment of intestinal barrier function due to the imbalance of the flora (50), which leads to the change of intestinal permeability, and the absorption of metabolites of the flora and endotoxins into the blood circulation in the body. These metabolites, including trimethylamine N-oxide (TMAO), bile acids, lipopolysaccharides, and short-chain fatty acids, all have an impact on the occurrence and development of atherosclerosis (51). These changes in intestinal flora and metabolites can not only cause coronary atherosclerosis, but even cause cerebrovascular diseases through gut-brain axis, inflammatory response, etc. (52), and even rupture of cerebral aneurysms in severe cases. Research has been reported that the *genus Campylobacter* and *Campylobacter ureolyticus* may be associated with the rupture of cerebral aneurysms, the gut microbiome profile of patients with stable unruptured intracranial aneurysms and ruptured aneurysms were significantly different (53). Secondly, the imbalance of intestinal fungi can also cause metabolic disorders. Some studies reported that compared with healthy lean individuals, the fecal fungi in obese participants showed more obvious diversity, and the intestinal fungal composition changed significantly. In addition, other studies have found that the abundance of *Thermoascus* and *species Malassezia restricta* in the patients with coronary atherosclerosis was significantly lower than in healthy individuals, and the decrease of *M.restricta* might have a close association with lipid metabolism disorder in atherosclerosis patients (46), there is growing evidence that antagonistic relationships between bacteria and fungi may reduce perturbations and enhance interactions in the gut, thereby establishing a balanced microbial community (54). Finally, a growing body of research also suggests that changes in enteroviruses are associated with cardiovascular disease, after an in-depth metatenomic analysis of the viriome of the participants’ fecal samples, the study found that enteroviruses in patients with cardiovascular disease were significantly different from healthy controls, for example, the *Siphoviridae* was significantly enriched in the viriome of patients with cardiovascular disease. In addition, the abundance of *Enterobacteriaceae* and *streptococcus* increased in patients with cardiovascular disease (36). As a result, the abundance of these viruses and bacteria presents a consistent level, in which the presence, absence, or abundance of viruses may regulate the progression of the disease by affecting bacteria in the host. Correlation analysis showed that *enterococcus*, *streptococcus* and *ruminococcus* were widely associated with viral operational taxonomic unit in patients with cardiovascular disease. This also reflects the fact that enteroviruses affect disease by relying on gut bacteria (47). In summary, we found that there may be a complex network among gut microbes, with interactions among bacteria, viruses, and fungi that jointly affect the occurrence and development of atherosclerosis.

The study has several advantages: MR analysis was used to establish the causal link between gut microbiota and AS, removing confounding variables’ involvement and lessening the effect on causal inference. Genetic variation in the gut microbiota was obtained from the largest available GWAS summary statistics, ensuring IVs strength in the MR analysis. The IVs selected in this study were all strong IVs (F > 10), which had high statistical power. By utilizing the MR-PRESSO and MR-Egger regression intercept term tests, horizontal pleiotropy was identified and excluded.

However, there are some limitations to this study. Because summary statistics were used in the analysis rather than raw data, we could not perform subgroup analyses, such as the analysis of gender differences. Since the lowest taxonomic level in the exposure dataset was genus, this limitation prevented us from further exploring the causal relationship between gut microbiota and AS at the species level. More genetic variants need to be included as IVs to perform sensitivity analyses and horizontal pleiotropy tests. Thus, the SNPs used in the analysis did not meet the traditional GWAS threshold for significance (*P* < 5×10 ^− 8^).

Due to confounding by ethnic stratification, data on gut microbiota were obtained from subjects of European ancestry, thus, the findings might not be entirely relevant to participants of non-European heritage. For greater generalization in the future, MR research on the causal link between gut microbiota and AS might be addressed in other populations.

## 5 Conclusion

In conclusion, this two-sample MR study found that some specific gut microbiotas were causally associated with the presence of AS. Further, RCT studies are needed to elucidate the protective or pathogenic mechanisms of probiotics or pathogenic bacteria in AS.

## Data availability statement

The original contributions presented in the study are included in the article/**Supplementary Material**. Further inquiries can be directed to the corresponding authors.

## Author contributions

SJ: Conceptualization, Formal Analysis, Software, Visualization, Writing – original draft. CY: Formal Analysis, Methodology, Software, Writing – review & editing. BL: Funding acquisition, Project administration, Supervision, Writing – review & editing. SH: Funding acquisition, Project administration, Supervision, Writing – review & editing. YZ: Methodology, Writing – review & editing. WY: Data curation, Methodology, Writing – review & editing. BW: Data curation, Funding acquisition, Investigation, Supervision, Writing – review & editing. DL: Conceptualization, Data curation, Investigation, Project administration, Writing – review & editing. JL: Conceptualization, Data curation, Funding acquisition, Project administration, Supervision, Visualization, Writing – review & editing.
