# Supplementary material for: Two-sample Mendelian randomization to study the causal association between gut microbiota and atherosclerosis
Source: Front Immunol. 2024 Jan 12;14:1282072. doi: 10.3389/fimmu.2023.1282072 (PMC10811052; doi:10.3389/fimmu.2023.1282072)
Supplement: Supplementary file 3 [file DataSheet_3.pdf]

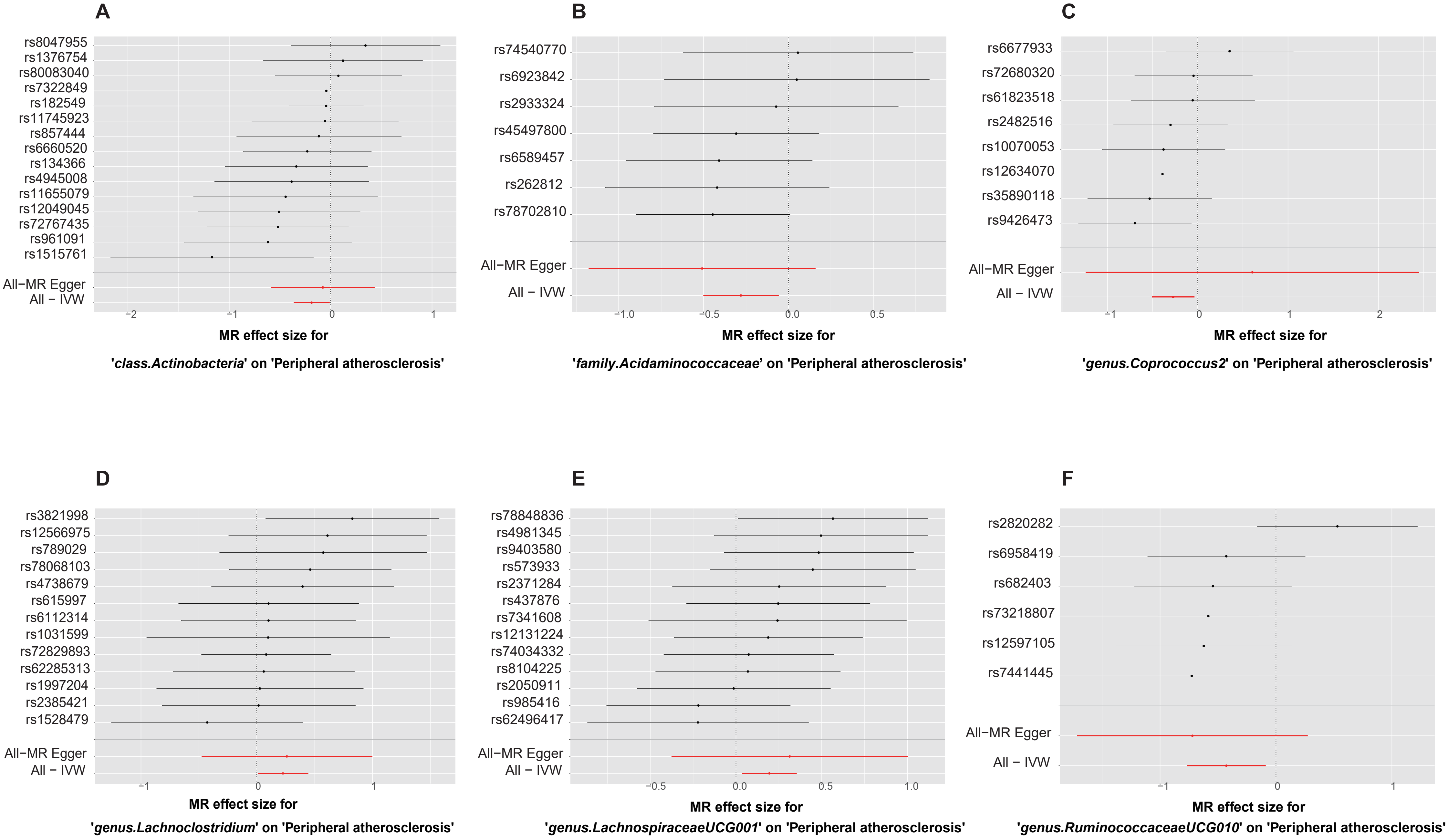

Forest plots for causal effects of gut microbiota on coronary atherosclerosis risk with individual SNPs.
